# Are Scoring Systems Useful in Predicting Mortality from Upper GI Bleeding in Geriatric Patients?

**DOI:** 10.3390/diagnostics15172173

**Published:** 2025-08-27

**Authors:** Mustafa Zanyar Akkuzu, Berat Ebik

**Affiliations:** Department of Gastroenterology, Diyarbakır Gazi Yasargil Education and Research Hospital, University of Health Sciences, Diyarbakır 21070, Turkey; beratebik@gmail.com

**Keywords:** upper GI bleeding, AIMS65 score, Rockall score, mortality, peptic ulcer, geriatrics

## Abstract

**Background/Objectives**: This study aimed to determine the in-hospital mortality rate after upper gastrointestinal (GI) bleeding in geriatric patients with comorbidities. Additionally, it sought to identify effective cut-off values for predicting high-risk patients using AIMS65 and Rockall scores and to assess the impact of oral anticoagulant and NSAID use on mortality. **Methods**: A retrospective cohort study was conducted on 64 patients aged 60 and above with at least one comorbidity who were admitted for upper GI bleeding between January 2023 and June 2024. AIMS65 and Rockall scores were calculated for each patient. The relationship between these scores, medication use, and mortality was analyzed using statistical methods, including ROC analysis and Kaplan–Meier survival curves. **Results**: The mean age was 77.6 years, and all patients had at least one chronic disease; 57.8% used medications increasing bleeding risk. In-hospital mortality was 18.7%, with no significant association for oral anticoagulants (*p* = 0.275) or NSAIDs (*p* = 0.324). Sepsis, heart failure, chronic renal failure, and malignancy were strongly linked to mortality in univariate analysis; multivariate analysis confirmed sepsis and malignancy as independent predictors, with a trend for heart failure. AIMS65 ≥ 2 (sensitivity 90.1%, AUC = 0.920) and Rockall ≥ 6 (sensitivity 91.7%, AUC = 0.822) were both effective in predicting mortality, with risk rising cumulatively with higher scores (*p* < 0.001). **Conclusions**: In-hospital mortality after upper GI bleeding is high in elderly patients with multiple comorbidities, mainly from sepsis, malignancy, and heart failure. AIMS65 and Rockall scores effectively predict mortality and may support earlier intervention. The small, high-risk cohort limits generalizability, underscoring the need for multicenter validation.

## 1. Introduction

Upper gastrointestinal (GI) bleeding is a potentially life-threatening condition. Although the overall incidence of non-variceal upper GI bleeding has decreased since the 1990s, the number of patients aged 65 and older has increased [1]. Approximately 70% of patients presenting with GI bleeding are over 60, and the incidence rises with age. Despite advances in diagnostic and therapeutic approaches, mortality remains high due to the aging population and the increased burden of comorbidities [2].

The use of oral anticoagulants and non-steroidal anti-inflammatory drugs (NSAIDs) in the treatment and prevention of cardiovascular events contributes significantly to the risk of ulcers and bleeding [2,3].

Scoring systems such as AIMS65 and Rockall have been developed to estimate the risk of mortality and rebleeding. However, there is no consensus on which scoring system is more effective. Moreover, while higher scores generally indicate increased mortality risk, specific cut-off values are not clearly defined [4].

This study aims to evaluate mortality in geriatric patients with upper GI bleeding and comorbidities, assess the predictive value of AIMS65 and Rockall scores for mortality, and identify cut-off values for these scores. The study also investigates the role of comorbidities, anticoagulant use, and NSAID use in mortality risk.

## 2. Material and Method

This retrospective cohort study included 64 patients admitted with upper GI bleeding between January 2023 and June 2024. Ethical approval was obtained from the ethics committee of Health Sciences University Diyarbakır Gazi Yaşargil Training and Research Hospital (2 October 2024, approval number 190).

### 2.1. Study Protocol

The study focused on patients over 60 with at least one comorbid disease, especially those chronically using oral anticoagulants or NSAIDs. A comparison group included patients with comorbidities but not using medications that increase bleeding risk.

All patients underwent guideline-recommended hemodynamic resuscitation, including cessation of oral intake and infusion of intravenous proton pump inhibitors. Erythrocyte transfusion was administered to maintain hemoglobin levels at 9–10 g/dL. Fresh frozen plasma was used for patients requiring more than 3 units of erythrocyte transfusion [5].

Endoscopy was performed within 6–36 h after stabilization. Peptic ulcer bleeding was classified per the Forrest classification [6]. Patients with high-risk ulcers (Forrest Ia, Ib, IIa, and IIb) received endoscopic hemostasis [7].

**Figure 1:**
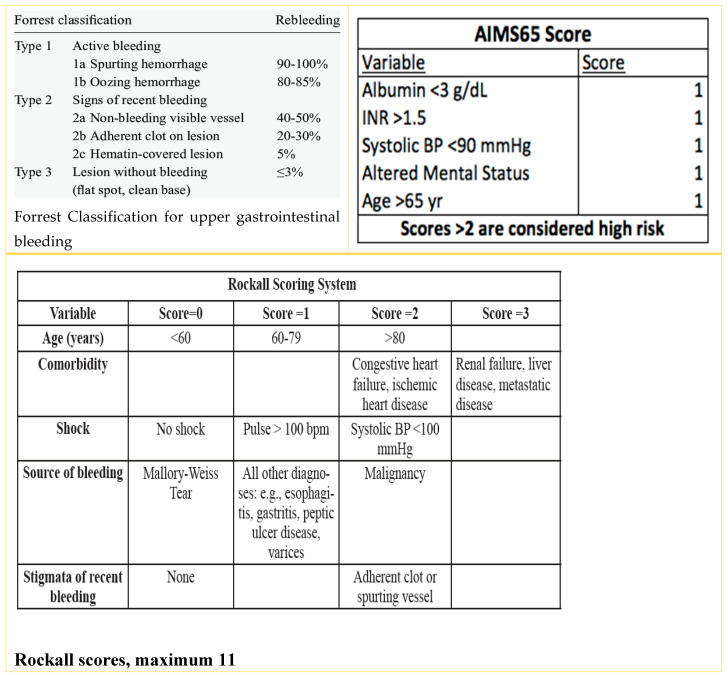
Forrest, AIMS65 score, and Rockall scoring systems used in GI bleeding.

Medication use was reviewed with prescribing specialists before making decisions regarding interruption. AIMS65 and Rockall scores were calculated for all patients. The Rockall score evaluates age, vital signs, comorbidities, and bleeding cause [8]. AIMS65 considers albumin, INR, mental status, blood pressure, and age [9] (Figure 1).

### 2.2. Exclusion Criteria

Patients with variceal bleeding, malignancy-related bleeding, other etiologies, those under 60, or those without comorbidities were excluded.

### 2.3. Patient Grouping

Patients were grouped by AIMS65 (<2 vs. ≥2) and Rockall (<6 vs. ≥6) scores. Mortality differences were analyzed. Additional grouping included patients on anticoagulants, NSAIDs, and those not on bleeding-risk medications.

### 2.4. Statistical Analysis

To check the normal distribution of patient data, Kolmogorov–Smirnov, Shapiro–Wilk test, coefficient of variation, skewness, and kurtosis methods were used. While mean and standard deviation values were stated in continuous variables, categorical variables were expressed as *n* (%). Independent T-test or Mann–Whitney U test was used to determine the medication use rates between recovered and exitus patients and to compare AIMS65 and Rockall scores between the groups. To determine the mortality of patients according to the drugs they used, one-way ANOVA was applied to groups with homogeneous variances, and Welch ANOVA and Kruskal–Wallis test were applied to groups with inhomogeneous variances. ROC analysis was performed to determine the sensitivity and specificity of Rockall and AIMS65 scores and the cut-off value. Kaplan–Meier survival analysis was performed to determine the cumulative effect of increases in both scores on mortality. Univariate and multivariate logistic regression analysis was performed to show the effect of comorbidities on mortality. All tests were two-sided, and a *p*-value < 0.05 was considered statistically significant. Statistical analyses were performed using the SPSS 24.0 for Windows (SPSS Inc., Chicago, IL, USA) package program.

## 3. Results

Among the 64 patients, 50% were female; the mean age was 77.6 (range 60–86). All had at least one chronic illness. The most common were hypertension (65.6%), diabetes mellitus (54.7%), chronic kidney disease (25%), and malignancy (defined as solid tumors including lung, colon, and breast cancers). A total of 78.1% had two or more comorbidities.

42.2% used no medications increasing bleeding risk; 39.1% used oral anticoagulants; 18.7% used NSAIDs. A history of hematemesis was present in 55.3%, previous GI bleeding in 33.9%, and in-hospital bleeding in 8.9%.

Forrest classification: Forrest 3 (54.7%), 2b (21.8%), 2c (9.3%), 2a (6.2%), 1b (6.2%), 1a (1.5%). All patients underwent endoscopy within a mean of 28.2 h. Bleeding control was achieved in all. A total of 54.7% received at least 3 units of blood (Table 1).

Mortality rates: NSAID users 8.3%, anticoagulant users 12.0%, non-users 29.6%. Differences were not statistically significant (NSAIDs *p* = 0.324; OACs *p* = 0.275; non-users *p* = 0.065).

Recovered patients had lower AIMS65 scores (2.40 ± 1.10 vs. 3.75 ± 0.75; *p* < 0.001). Among the recovered, 25.0% had AIMS65 ≥ 2; among the deceased, 91.6% (*p* = 0.001). Similar findings applied to Rockall scores (4.98 vs. 8.75; *p* < 0.001). Rockall ≥ 6: 26.9% in recovered vs. 83.3% in deceased (*p* = 0.001) (Table 2).

**Figure 2:**
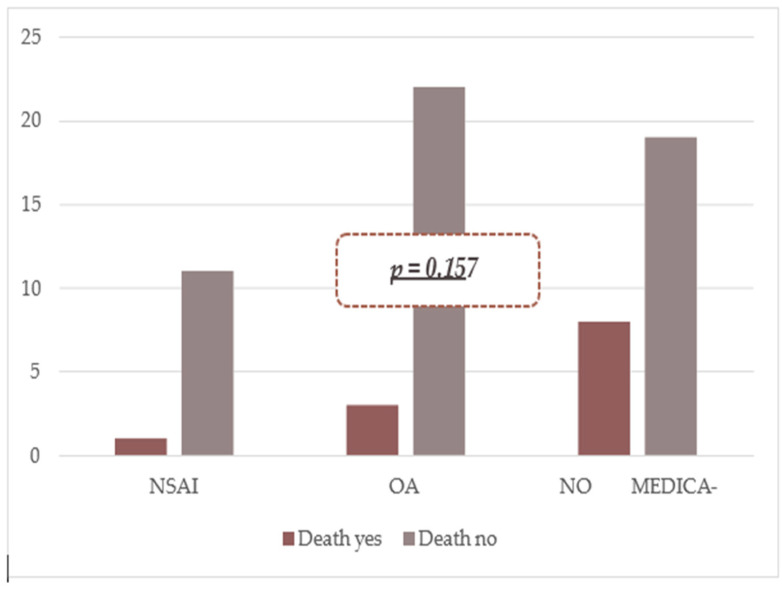
Mortality rates according to the drugs used by patients with GI hemorrhage.

When patients were categorized into three groups based on medication use, no significant mortality difference was observed (*p* = 0.157) (Figure 2).

**Figure 3:**
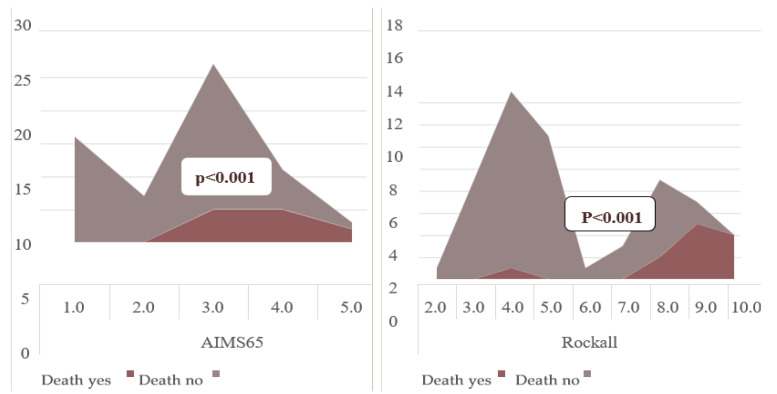
The relationship between AIMS65 score and Rockall score and mortality in patients with GI bleeding.

**Figure 4:**
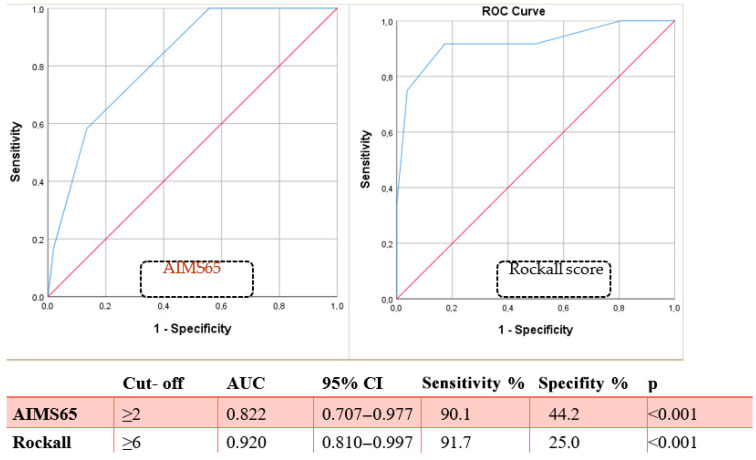
ROC analysis showing the cut-off values predicting mortality in AIMS65 and Rockall score. The red line is the standard mean line found in ROC analyses. The blue line indicates the AUC area.

ROC analysis: AIMS65 ≥ 2 predicted mortality with 90.1% sensitivity and 44.2% specificity (AUC = 0.920; *p* < 0.001). Rockall ≥ 6 had 91.7% sensitivity and 25% specificity (AUC = 0.822; *p* < 0.001). Kaplan–Meier analysis showed cumulative mortality increased with rising scores (*p* < 0.001) (Figure 3 and Figure 4, Table 2).

Most deaths occurred in patients with Forrest 3 ulcers who required minimal transfusion (1–2 units). These deaths were largely due to decompensation from underlying chronic diseases such as cardiac failure and malignancy-related complications, not active bleeding. A total of 7.8% of patients died due to sepsis, 4.7% due to decompensated heart failure and cardiac disease, 4.7% due to malignant disease, and 1.5% due to chronic renal failure.

When we examined the effect of comorbid diseases on mortality in elderly patients with peptic ulcer bleeding, univariate analysis determined that sepsis was a highly predictive factor on mortality (OR: 10.25; *p* = 0.004). In addition to sepsis, heart failure (OR: 5.00; *p* = 0.011), chronic renal failure (OR: 3.83; *p* = 0.023), and malignancies (OR: 4.64; *p* = 0.017) also had independent predictive effects on mortality. Multivariate analysis also showed that sepsis (OR: 6.46; *p* = 0.009), heart failure (OR: 3.18; *p* = 0.06), and malignancies (OR: 2.77; *p* = 0.040) had a cumulative effect on mortality in the geriatric patient group with peptic ulcer bleeding (Table 3).

**Figure 5:**
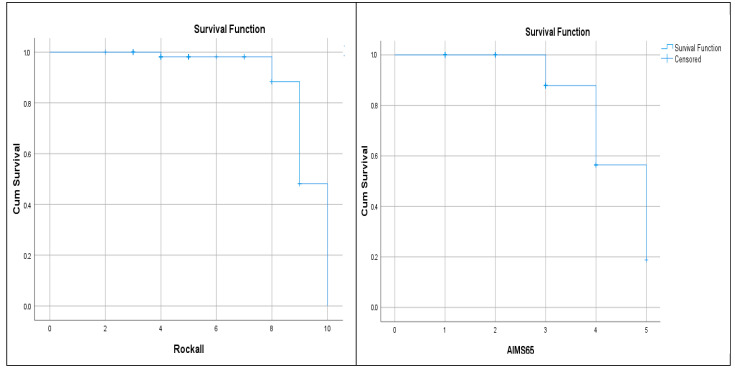
Kaplan–Meier analysis of cumulative increase in mortality as Rockall and AIMS65 scores increase.

As a result of statistical analysis, the AIMS65 and Rockall scores were found to be statistically significant in predicting in-hospital mortality in our study (*p* < 0.001). When the two scores were compared, the AIMS65 and Rockall scores were not statistically superior to each other in predicting in-hospital mortality. In addition, it was seen that both the AIMS65 and Rockall scores increased mortality in GI bleeding cumulatively (*p* < 0.001) (Figure 5). Mortality increased significantly as the score points increased.

## 4. Discussion

It is important to identify patients who may need intensive care and early endoscopic intervention for upper GI bleeding [10]. Because a scaling system that can detect risky patients earlier and perform effective triage can contribute to reducing mortality in this patient group [11,12].

Although all patients with upper GI bleeding present to the emergency department with a common symptom such as hematemesis or melena, the clinical course and prognosis of the patients may be completely different. In this case, the algorithm or scoring system to be used should be able to distinguish between patients and determine which patients need to be hospitalized in intensive care, as well as patients who need early endoscopy. The AIMS 65 score includes parameters such as hypotension, which is an important predictor of severe bleeding, as well as age, which is an important marker that increases mortality, and albumin levels [13], which are indicators of chronic diseases and therefore serious comorbid diseases. This shows that the AIMS65 score can be effective in identifying patients at risk at the time of first admission to the emergency department [14].

Similarly, the Rockall score uses age and blood pressure as parameters, as does the AIMS 65 score [15]. Although there is no albumin level in Rockall, there is a tab where chronic diseases are scored instead. Heart failure, chronic renal failure, and malignancies that may directly increase the patient’s mortality are scored [16]. Both scoring systems can be used at first presentation, and each increasing score allows for early triage of patients with GI bleeding.

The in-hospital mortality rate observed in our cohort of elderly patients with upper gastrointestinal (GI) bleeding and comorbidities was 18.7%, which is notably higher than rates typically seen in younger populations. No deaths were directly due to uncontrolled hemorrhage; most were related to decompensation from sepsis, heart failure, or malignancy. Univariate and multivariate analyses confirmed sepsis, malignancy, and (with borderline significance) heart failure as independent predictors of mortality. These findings underscore the multifactorial nature of outcomes in this demographic and suggest that specific comorbidities—not just overall comorbidity count—should be considered when assessing prognosis. Notably, many of the deceased patients had minimal transfusion needs, further supporting a non-hemorrhagic mechanism of death.

Interestingly, no statistically significant association was found between oral anticoagulants (OACs) or non-steroidal anti-inflammatory drugs (NSAIDs) use and mortality, and these findings should be interpreted with caution due to wide confidence intervals and limited statistical power. While our findings contrast with several large-scale studies and meta-analyses that suggest [17,18,19] an elevated mortality risk in anticoagulated patients, these discrepancies may be explained by our relatively small sample size, early intervention strategies, or the exclusion of variceal and malignancy-related bleeding cases in our cohort.

Both the AIMS65 and Rockall scoring systems proved to be effective in predicting in-hospital mortality. Patients with an AIMS65 score ≥ 2 or a Rockall score ≥ 6 exhibited significantly higher mortality. These cut-off values correspond with those proposed in other studies [20,21]. The predictive accuracy of AIMS65 may be partially attributable to its inclusion of albumin, a known independent predictor of mortality [22]. Rockall, on the other hand, accounts for age, hemodynamic instability, and comorbidity burden, all of which are relevant in this population.

Kaplan–Meier analysis showed a dramatic increase in mortality in those with a Rockall score of 8 or higher. Similarly, the same analysis showed that when the AIMS65 score exceeded 4, the increase in mortality became evident. Although previous studies did not provide clear cut-off values for these scoring systems [11,23], in our study we were able to obtain data that could suggest both basal and critical cut-off levels for both scoring systems.

Our findings align with the prior literature: Hyett et al. [24] found AIMS65 superior in predicting mortality, whereas the Glasgow–Blatchford Score (GBS) was better suited for predicting transfusion requirements. Uçmak et al. and Robertson et al. also reported that AIMS65 and Rockall were more reliable than GBS in forecasting clinical outcomes [25,26]. Other studies, such as those by Chang, Kim, and Jung, have found either no significant difference or variable performance depending on the patient population and endpoint [27,28,29]. Furthermore, these results are consistent with previous findings in colorectal cancer patients, where bleeding location and etiology significantly influence outcomes [30].

Although a slightly higher AUC was found for Rockall compared to AIMS65 in our study (0.920 vs. 0.822), the difference was not statistically significant. Both scores were comparably effective overall, especially when applied in the early stages of patient evaluation. Importantly, our study adds novel data by linking specific high-risk comorbidities (sepsis, malignancy, heart failure) to mortality risk in elderly patients with peptic ulcer bleeding.

Crucially, this study is one of the few to simultaneously assess the effects of bleeding-related medications and risk scores in a geriatric population. Our results suggest that while medication use alone may not significantly alter outcomes, the integration of structured risk stratification using AIMS65 and Rockall scores can yield more precise, individualized management strategies.

From a diagnostic standpoint, these scoring systems serve not only as prognostic tools but also as integral components of clinical decision-making. Their utility in emergency departments, particularly for identifying high-risk patients and guiding early endoscopy or ICU admission, has implications for resource allocation and workflow optimization. Furthermore, there is growing potential to embed these tools within electronic medical record systems and clinical decision support (CDS) platforms, thus enhancing diagnostic efficiency and consistency.

Emerging applications in artificial intelligence (AI) and machine learning could also benefit from these structured data points. Integrating AIMS65 and Rockall parameters into predictive models may support the development of real-time, algorithm-based triage systems, which are particularly valuable in high-volume or resource-limited settings.

Although the sample size was small, it reflects the strict inclusion of a clinically high-risk population—elderly patients (≥60 years) with peptic ulcer bleeding and multiple comorbidities. The elevated mortality observed is likely attributable to this specific risk profile rather than to a broader, lower-risk population.

## 5. Limitations

This single-center study included a relatively small sample (*n* = 64), which limits statistical power and generalizability. However, the cohort represents a strictly defined, clinically high-risk group—elderly patients with peptic ulcer bleeding and multiple comorbidities—explaining both the small number and the high mortality observed. While comorbidities of varying severity were initially grouped together, additional subgroup analyses identified sepsis, malignancy, and heart failure as major independent predictors of mortality. The retrospective design may introduce bias, and larger multicenter studies are needed to confirm these findings.

## 6. Conclusions

Mortality rates remain significantly high following upper gastrointestinal (GI) bleeding, particularly in elderly patients with multiple comorbidities. Although the cohort size was limited, this reflects a strictly defined high-risk population—elderly patients with peptic ulcer bleeding and substantial comorbidity burden—which increases the clinical relevance of the findings despite reduced generalizability. This study reinforces that early and accurate risk stratification plays a pivotal role in clinical outcomes, especially in this vulnerable population. Our findings indicate that the use of oral anticoagulants or non-steroidal anti-inflammatory (NSAID) drugs does not independently increase in-hospital mortality; however, this should be interpreted cautiously due to the wide confidence intervals and limited statistical power. This insight challenges earlier assumptions and underscores the importance of individualized risk assessment rather than blanket risk attribution to these medications. It also shifts the diagnostic emphasis from medication history alone to integrated scoring and clinical evaluation.

Critically, both the AIMS65 and Rockall scoring systems emerged as powerful diagnostic tools for predicting in-hospital mortality. These scores utilize readily available clinical and laboratory data—such as albumin levels, systolic blood pressure, and comorbid burden—to deliver real-time risk assessments. In a diagnostic setting, this translates into faster triage, targeted use of intensive care resources, and more informed decisions regarding early endoscopic intervention. Moreover, the growing interest in machine learning–based diagnostic models in gastroenterology could benefit from incorporating AIMS65 and Rockall variables as training features. This may lead to the development of more sophisticated, real-time decision algorithms that support physicians in fast-paced clinical environments.

In conclusion, this study highlights not only the prognostic value of scoring systems in managing GI bleeding but also their emerging role as diagnostic instruments that guide the clinical pathway from emergency triage to definitive care. By embedding validated risk models into diagnostic algorithms, we can significantly enhance the safety, speed, and precision of care delivered to elderly patients with upper GI bleeding.

## Figures and Tables

**Table 1:** Demographic, laboratory, and clinical data of patients with GI bleeding, along with the medications they used and bleeding characteristics.

Age (Years)	77.6 ± 8.7 (60–86)	
Gender (*n*/%)	F/M 32 (50%)/32 (50%)	
Comorbid diseases	HT	42 (65.6%)
	DM	35 (54.7%)
	HF	26 (40.6%)
	CKD	15 (23.4%)
	Malignancy	11 (17.2)
	Neurological diseases	9 (14.1)
	Chronic lung diseases	4 (6.2%)
	Chronic liver disease	7 (10.9)
Drug Use	None	*n*: 27 (42.2%)
	Anticoagulants or DOACs	*n*: 25 (39.1%)
	NSAIDs	*n*: 12 (18.7%)
Forrest classification	Forrest 3: 54.7%	
	Forrest 2c: 9.3%	
	Forrest 2b: 21.8%	
	Forrest 2a: 6.2%	
	Forrest 1b: 6.2%	
	Forrest 1a: 1.5%	
Causes of death	Sepsis: 7.8%	
	Decompensated heart failure and cardiac disease: 4.7%	
	Malignant disease: 4.7%	
	Chronic renal failure: 1.5%	

HT: hypertension; DM: diabetes mellitus; HF: heart failure; CKD: chronic kidney disease; NSAIDs: non-steroid anti-inflammatory drugs; DOACs: direct acting oral anticoagulants. Note: Malignancies were lung cancer, breast cancer, hepatocellular cancer, and colon cancer.

**Table 2:** Mortality risk of patients who recovered and death as a result of upper GI bleeding according to the drug group they used and the mean AIMS and Rockall scores.

	Recovered (*n* = 52)	Death (*n* = 12)	*p*-Value	OR (95% CI)
NSAID	11 (91.7%)	1 (8.3%)	0.324	0.33 (0.3–2.9)
OAC	22 (88.0%)	3 (12.0%)	0.275	0.45 (0.1–1.8)
Does Not Use Medication	19 (70.4%)	8 (29.6%)	0.065	3.47 (0.9–13.0)
AIMS65 (mean)	2.40 ± 1.10	3.75 ± 0.75	<0.001	
AIMS65 (≥2)	13 (25.0%)	11 (91.6%)	0.001	33.0 (3.8–136.5)
Rockall (mean)	4.98 ± 1.82	8.75 ± 1.65	<0.001	
Rockall (≥6)	14 (26.9%)	10 (83.3%)	0.001	13.5 (2.6–49.7)

**Table 3:** Univariate and multivariate logistic regression analysis showing the effect of existing comorbidities on mortality in elderly patients who died from upper peptic ulcer bleeding.

	Univariate Analysis			Multivariate Analysis		
Parameter	OR	95% CI	*p*	OR	95% CI	*p*
HT	0.44	0.32–1.59	0.212			
DM	0.79	0.33–2.78	0.717			
Sepsis	10.25	2.94–25.13	0.004	6.46	1.50–10.89	0.009
Heart failure	5.00	2.26–11.69	0.011	3.18	1.24–7.63	0.026
Chronic liver disease	0.69	0.09–6.40	0.749			
Chronic kidney disease	3.83	0.98–10.58	0.023	1.99	0.99–5.01	0.051
Chronic lung disease	0.53	0.27–9.63	0.683			
Malignancy	4.64	1.23–12.49	0.017	2.77	1.17–6.85	0.040
Neurological diseases	0.51	0.05–4.32	0.546			

## Data Availability

The datasets used and/or analyzed during the current study are available from the corresponding author upon reasonable request.
